# Comparison of commercial nanoliquid chromatography columns for fast, targeted mass spectrometry-based proteomics

**DOI:** 10.4155/fsoa-2016-0014

**Published:** 2016-03-16

**Authors:** Tore Vehus, Kristina Erikstad Seterdal, Stefan Krauss, Elsa Lundanes, Steven R Wilson

**Affiliations:** 1 Department of Chemistry, University of Oslo, P.O. 1033 Blindern, NO‐0315 Oslo, Norway; 2 Unit for Cell Signaling, SFI-CAST Biomedical Innovation Center, Oslo University Hospital, Rikshospitalet, NO‐0027 Oslo, Norway

**Keywords:** Chromatography, mass spectrometry, parallel reaction monitoring, SILAC, targeted proteomics

## Abstract

**Aim:**

We compared four commonly used, commercially available reverse phase nanoLC columns for identification/determination of Wnt/β-catenin-related pathway proteins.

**Materials & methods:**

The columns were: Chromolith^®^ (silica monolith; Merke Millipore, MA, USA), PepMap™ (porous particles; Thermo Fisher Scientific, MA, USA), Accucore™ (solid core particles; Thermo Fisher Scientific) and PepSwift™ (organic monolith; Thermo Fisher Scientific).

**Results:**

The peak capacity of the columns varied from 100 (Pepswift) to 190 (Accucore) (for 30 min gradients). All columns enabled identification/detection of GSK3β and β-catenin in the complex samples. However, even the columns with higher peak capacities could not enable detection of the somewhat less abundant proteins AXIN2 and TNKS2. The monoliths were more prone to retention time instability when sample complexity increased.

**Conclusion:**

We find that commercial nanoLC columns, although featuring different morphologies and peak capacities, provided surprisingly few practical differences for relatively fast, targeted determination of proteins.

## Abstract (lay abstract)

**Lay abstract:** The measurement of proteins (which can indicate diseases) in biological samples is commonly done with nonspecific approaches (can give rise to false results) that can be tedious and time consuming, especially when many proteins are to be measured from the same sample. Nanoliquid chromatography-mass spectrometry is an alternative approach that is very specific, and can be used to measure many proteins at once. There are several variants of nanoLC (i.e., nanoLC columns that are connected to the MS), but we have found that it is rather indifferent which nanoLC column is used for fast analysis. Hence, new nanoLC variants are called for to push the technique further.

**Figure 1.**
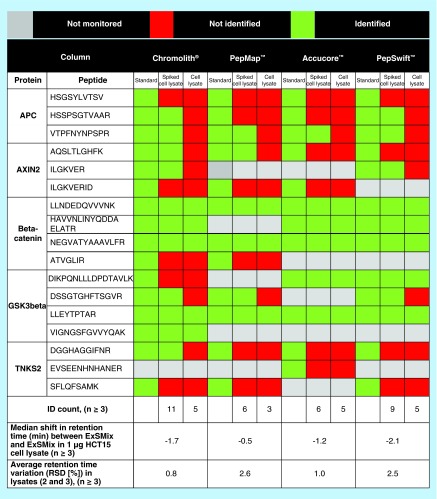
**Peptide identification for each chromatographic column investigated.** Standard is 0.5 ng ExSMix, Spiked cell lysate is 0.5 ng ExSMix spiked into 1 µg HCT15 tryptic cell lysate and Cell lysate is 1 µg HCT15 tryptic cell lysate (all in triplicates). Green equals identified, red not identified and gray not observed with ddMSMS. MS/MS extraction was done with 7 ppm mass accuracy. Minimum three transitions were required for positive identification and not more than 0.5 min shift in t_R_ between 2 and 3 were allowed for positive identification. At least three injections per group were used. ddMSMS: Data-dependent MS/MS; RSD: Relative standard deviation.

**Figure 2.**
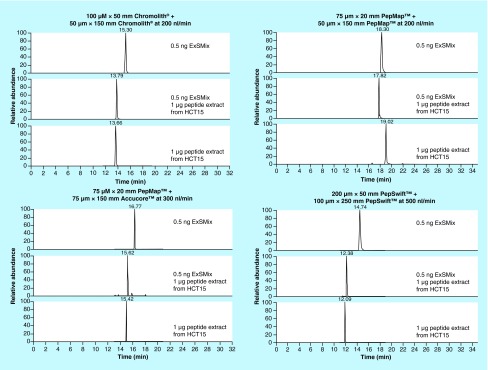
**EIC of LLNDEDQVVVNK corresponding to β-catenin in 0.5 ng ExSMix, 0.5 ng ExSMix spiked in 1 µg tryptic peptide extract from HCT15 and 1 µg tryptic peptide extract from HCT15 for each of the chromatographic systems investigated.** (Chromatographic conditions and MS-settings as described in ‘Materials & methods’).

**Figure 3.**
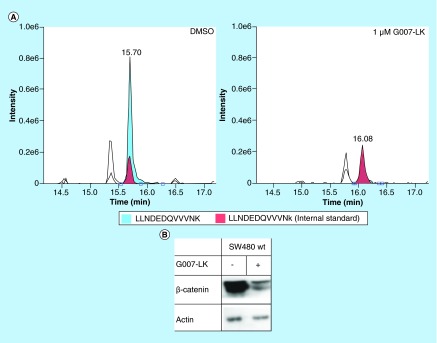
**(A)** EICs of LLNDEDQVVVNK (blue color, endogenous) and LLNDEDQVVVNk (red color, internal standard) in Dimethylsulfoxide and in 1 µM G007-LK treated SW480 cells, respectively, analyzed by LC-MS/MS on the Accucore™ (Thermo Fischer Scientific, MA, USA) column using standard gradient conditions and MS/MS detection in duplexing mode with resolution at 35,000 and maximum injection time at 100 ms. **(B)** Western blot of total β-catenin and actin in Dimethylsulfoxide and 1 µM G007-LK-treated SW480 cells. (Procedure in Supplementary Materials & Methods).

Targeted proteomics with nanoLC-ESI-MS/MS can be used to selectively detect/quantify specific proteins, often within short analysis times compared with more comprehensive approaches (e.g., 30 min instead of several hours) [1–3]). Operating the MS in selected/parallell reaction monitoring (SRM/PRM) [4] increases sensitivity in targeted proteomics. However, high performance separation columns are also required to resolve compounds prior to MS detection, reducing ion suppression during the electrospray process. A key descriptor of LC resolution is the peak capacity of the column (i.e., the number of compounds that can be chromatographically separated) [5,6]. Several variants of nanoLC columns can provide high peak capacity, for example, columns packed with solid core particles or totally porous particles, and silica-based monoliths and organic monoliths [7–9]. All of these column variants can provide high-resolution separations for comprehensive proteomics [9–12]. Similar columns have previously been partly compared regarding peak capacity, evaluated with simple standards [13], but a comparison for ‘real-life’ proteomics (e.g., relatively fast gradients, very complex samples), with a focus on column robustness (performance with standards vs complex samples), has not been performed.

We wished to investigate commercially available columns with the above-mentioned morphologies regarding performance for determination of central proteins of the cancer-associated Wnt/β-catenin pathway [14] (a key focus of our research [15,16]). These proteins are present at moderate–low concentrations (60 – <1 ng/µl). Specifically, peak capacity, peak shape, carryover and retention time repeatability were assessed.

## Materials & methods

### Sample preparation

Recombinant APC (H00000324-Q01) and AXIN2 (H00008313-Q01) were purchased from Abnova (Tapei City, Taiwan). Glycogen synthase 3β (GSK3β) were from Life Technologies (CA, USA) and β-catenin (12–537) from Millipore (MA, USA). The poly-ADP-ribosylation polymerization (PARP)-domain of human tankyrase2 (TNKS2) was produced as described in [17]. All amino acid sequences can be found in Supplementary Table 1.

Each standard protein was digested with trypsin (Promega, Madison, WI, USA). Briefly, 10 µg of each protein was dissolved in 1 ml 8 M urea (Sigma Aldrich) dissolved in 50 mM Tris-HCl pH 8.0 (Sigma Aldrich). The samples were reduced in 5 mM dithiothretiol (Sigma Aldrich) at 37°C for 30 min and alkylated with 15 mM iodoacetamide for 15 min in the dark. The urea concentration was reduced to 1.5 M by adding 50 mM Tris-HCl pH 8.0. Trypsin was added to a protein:enzyme ratio of 1:20, and incubated over night at 37°C. The digested standards were desalted using solid phase extraction (SPE) on reversed phase (RP) C_18_ cartridges (Bond Elut C_18_, 100 mg, Agilent, CA, USA) with water (Millipore) and eluted in 1 ml 80% acetonitrile (ACN, HiPerSolv CHROMANORM^®^, VWR, Radnor, PA, USA) with 0.1% formic acid (FA, Sigma Aldrich) (v/v) and dried with a SpeedVac (Thermo Fischer Scientific; former Savant, MA, USA). Each standard were reconstituted in 0.1% (v/v) TFA (Sigma Aldrich) to a final concentration of 10 µg/ml.

A set of external standard mixtures (ExSMix) containing 1, 0.5, 0.1, 0.05, 0.01, 0.005, 0.001, 0.0005 and 0.0001 µg/ml of each protein standard were prepared by appropriate dilution with 0.1% (v/v) TFA.

HCT15 cells (American Type Culture Collection, ATCC, VA, USA) were cultured in RPMI 1640 medium (Life Technologies) supplemented with 10% fetal bovine serum (Life Technologies) and penicillin streptomycin (Life Technologies) and harvested with trypsin EDTA (Life Technologies) at 80% confluence. The cells were counted and washed in phosphate-buffered saline (Oslo University Hospital, Oslo, Norway). The proteins were extracted and digested using the filter-aided sample preparation protocol [18]. Briefly, 1 million cells were resuspended in 200 μl lysis buffer, heated for 15 min at 70°C and sonicated for 5 min. Debris was removed with centrifugation at 13,000 rpm for 10 min in a thermostated centrifuge at 20°C (Eppendorf, Hamburg, Germany). The protein concentration was determined using Bradford Assay (Bradford Quick Start Assay, Bio-Rad, CA, USA) at absorbance of 595 nm with bovine serum albumin (Sigma Aldrich) used as calibration standards. About 100 μg protein was added to 10 kDa 0.5 ml filter devices (Millipore). The filter-aided sample preparation two-step digestion protocol with Trypsin-LysC mix (Promega) (1:20 protein:enzyme ratio) was followed [18]. The peptides were desalted using the above-described SPE-procedure. Peptide concentrations were determined using a NanoDrop2000 instrument (Thermo Fisher Scientific) with absorbance at 205 nm with 31 mg/ml absorption coefficient. The samples were diluted to a final concentration of 1 mg/ml with 0.1% (v/v) TFA.

An internal standard protein solution (IS_prot_) was prepared by SILAC labeling of HEK293 (ATCC) and HCT15 cells according to the procedure described by Ong & Mann [19] with ^13^C_6_^15^N_4_-arginine and ^13^C_6_-lysine (+10.008 and +6.020 Da, respectively) (Thermo Fisher Scientific) supplemented to RPMI1640 Media for SILAC acquired from Thermo Fisher Scientific. The labeled cell lines were subsequently lysed as described above and added to samples prior to protein digestion. Heavy amino acid incorporation was verified with data-dependent LC-MS/MS analysis of nonlabeled cell lines and labeled cell lines (data not shown).

### Treatment of cell lines with G007-LK

The colon carcinoma cells were seeded (100,000 cells/well) in 6-well plates (Nunc™ Cell-Culture Treated Multidishes, Thermo Fisher Scientific). RPMI1640 were used for HCT15 and COLO320DM (ATCC) cells with incubation in 5% CO_2_, and Leibowitz L‐15 medium (Thermo Fisher Scientific) for SW480 (ATCC) cells with incubation in 0% CO_2_. After 24 h, the medium was removed and the tankyrase inhibitor G007-LK [17] (dissolved in dimethylsulfoxide (Sigma Aldrich) was added to a final concentration of 1 μM in the cells’ respective medium. An equal volume of dimethylsulfoxide was added as negative control. After 24 h of incubation the cells were harvested and processed as described above.

Three biological replicates were made and analyzed, with exception of COLO320DM and SW480 cells, were treated cells were analyzed in duplicates.

### LC instrumentation

A NanoLC1000 pump from Thermo Fisher Scientific was used in this study. The mobile phase A (MP A) contained 0.1% (v/v) FA in H_2_O (Optima^®^ LC/MS, Fisher Scientific, part of Thermo Fisher Scientific) and mobile phase B (MP B) contained 0.1% (v/v) FA in ACN. The pre- and analytical columns were coupled through a stainless steel T-piece (Valco, VICI AG International, Schenkon, Switzerland). Each gradient of 30 min was followed with a linear increase to 95% MP B for 10 min and a 10–15 min hold at 95% MP B. Each pre- and analytical column was equilibrated with at least six column volumes.

The Acclaim^®^ PepMap RSLC (PepMap™) 75 μm × 20 mm and 50 μm × 150 mm particle packed pre- and analytical columns, the PepSwift^®^ 200 μm × 5 mm and 100 μm × 250 mm monolithic poly-styrene divinylbenzene (PS-DVB) pre- and analytical columns, the Accucore™ 75 μm × 150 mm solid core particle packed column were from Thermo Fisher Scientific and the 100 μm × 50 mm and 50 μm × 150 mm Chromolith^®^ Caprod^®^ silica monolithic pre- and analytical columns were from Merck-Millipore.

### MS & MS/MS parameters

The electrospray voltage was set to 1.8 kV for the 2 μm inner diameter (ID) stainless steel emitter (Thermo Fisher Scientific), the 5 and 8 μm ID New Objective Emitters (New Objective, Woburn, MO, USA). The Accucore and PepSwift were connected to the MS through PicoTip ESI emitters fitted for the column flow rate used. The PepMap and Chromolith columns were connected to stainless steel emitters.

A Q Exactive™ Hybrid Quadrupole-Orbitrap mass spectrometer (Thermo Fisher Scientific) was used for the entire study. Two main methods were used, full-MS with subsequent data-dependent MS/MS (ddMSMS) and targeted-MS/MS with selected ions from the proteotypic peptides chosen. In full-MS, the resolution was set to 140,000 at m/z 200, automatic gain control (AGC) to 1,000,000, maximum inject time to 100 ms and scan range m/z 350–1850. The 10 most intense ions were selected and fragmented using normalized collision energy (NCE) of 25% and the MS/MS scans were acquired with; resolution of 70,000, AGC target value of 100,000, and maximum inject time of 500 ms. Dynamic exclusion was enabled for 20 s, and charge states of 1 and >7 were rejected for fragmentation.

For targeted MS/MS, each target was monitored in a retention time window of ±4 min relative to the retention time determined by ddMSMS, and either operated in single or duplexing mode. For single ion fragmentation, the maximum injection time was set to 500 ms, with an AGC target value of 100,000, and a resolution of 140,000. The isolation width was set to m/z 4.0 and the NCE to 25 eV.

In duplex mode, the resolution was decreased to 35,000, the maximum inject time lowered to 100 ms and the AGC target value kept unaltered. Each target was fragmented with an isolation of m/z 2.0 and NCE of 25 eV.

### Peptide identification & proteotypic peptide selection

Using the Skyline software (v2.5) [20], a theoretical tryptic digest of each protein standard was made and together with data-dependent LC-MS/MS runs for each column, a set of proteotypic peptides was selected for each protein (Supplementary Table 2). The peptides selected were checked for uniqueness against the Uniprot database [21] and only peptides selectively representing the proteins of interest were chosen as proteotypic peptides. Peptides containing cysteine, methionine, serine, tyrosine and threonine were avoided if other possibilities were available. A minimum of 3 m/z transitions above 350 m/z were chosen (Precursor and fragment m/z are shown in Supplementary Table 2).

### Data processing

All chromatograms were analyzed with Xcalibur software (v2.1, Thermo Fisher Scientific) and Proteome Discoverer (v1.4, Thermo Fisher Scientific) with the Sequest algorithm were used for searching against the protein sequences found in Supplementary Table 1 and Supplementary Excel File, and against the Uniprot database for comprehensive identification. Peak capacity was measured for at least 5 peptides manually with a confidence of ± 0.01 min. Peak widths, heights and areas were measured manually, and peak capacity (n_c_) at half peak height was calculated according to Equation 1,
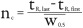



where 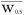 is average peak width at half peak height, t_R, first_ and t_R, last_ are the retention times for the first and last eluting peak, respectively.

#### Data-dependent MS/MS

For identification in ddMSMS of complex samples, maximum two missed cleavages, maximum 10 ppm precursor and 0.6 Da mass tolerance, respectively, were allowed, combined with a false discovery rate of 0.01. A minimum of two peptides, where at least one had to be unique were used for positive identification. Carbamidomethylation was set as a static modification and oxidation of methionine as dynamic. When labeled samples were processed, heavy lysine and arginine were set as fixed modifications.

#### Targeted MS/MS

Targeted MS/MS data acquired at resolutions of 140,000 and 35,000 were extracted using 7 and 25 ppm, respectively. Identification of proteins in targeted MS/MS was done manually with Xcalibur software.

### Statistical analysis

Optimization of flow rate in terms of measured peak capacity was based on one injection per flow rate, and measured for >5 peptides eluting throughout the gradient (Supplementary Figures 1–6). For the optimal separation conditions, the values are based on at least three injections, and more than five peptides. Peak capacity relative variation was less than 10%. Identifications in complex samples were based on at least three replicates.

For statistical analysis, F-test and two-sided student t-test were used, where n ≥ 3.

## Results & discussion

### Framework

The RP columns investigated were Chromolith CapRod (C_18_ silica monolith), PepMap (C_18_ porous particles), Accucore (C_18_ solid core particles) and PepSwift (PS-DVB organic monolith). These represent the most common used columns/morphologies in modern bottom-up proteomics. The columns available had some variations in dimensions (50–100 µm IDs and 15–25 cm length); identical dimensions were not commercially available for all columns, but at optimum conditions the t_G_/t_0_ were almost identical (see below) and comparison of performance could be done, using the procedure described by Wang *et al.* [6]. The linear separation gradient was set to 30 min (= t_G_), with a total analysis time of 1 h (including washing steps, equilibration and sample loading); this was considered to be an acceptable compromise between speed and risk of ion suppression. For each column, the gradient was adjusted so that the last eluting peptide of interest eluted at t_G_, One column per instrument maker was investigated; batch to batch variations of these columns is minimal (2–5% variation [22,23]). A standard mobile phase consisting of water, 0.1% FA, and ACN were used for all columns. Proteins studied were AXIN2, β-catenin,GSK3β and TNKS2, which are key targets in our efforts in developing novel cancer therapeutics [15]. Additionally, the C-terminal of APC was included, and served as a negative control for protein identification in the APC-mutated colon carcinoma cell lines. The amounts of complex samples loaded onto each system were between 0.5 and 1 μg, amounts that are common in proteomics experiments [24,25] and well below the precolumn capacities reported by the manufacturers. The LC-MS/MS system set-up was considered ‘healthy’ as 40 proteins/separation minute could be identified in comprehensive mode (Supplementary Excel File).

### Comparison of column performance, using a standard mixture

To compare the nanoLC columns’ performance for 30 min gradients, the flow rate and gradient composition were optimized for each column with regards to peak capacity according to the procedure described by Wang *et al.* [6]. The definition used for peak capacity is found in Eq.1 (Materials and methods) A key point is to fully exploit the separation window, ensuring that the most hydrophobic analytes elute at the end of the gradient (t_final peak_ = t_G_). The sample was the ExSMix (tryptic peptides from recombinant APC, AXIN2, β-catenin, GSK3β and tankyrase2, see Materials and methods). Table 1 shows the peak capacity and peak asymmetry for each column set-up, as well as the enabling solvent conditions. The highest peak capacity was obtained with the solid core particle packed column set-up, with an average peak capacity close to 190 (Supplementary Figure 7); approximately 1.5-times larger compared with the second-best performing column (the silica monolith, peak capacity = 130) with our conditions. The solid core particle packed based column also provided the least peak tailing and the narrowest peaks of the columns included in this study, with an average asymmetry of 1.1 and a base peak width of 10 s. The two monolithic columns tested displayed more peak tailing compared with the particle-based columns (1.4–1.5 vs 1.1–1.3). The optimal linear gradient composition was the same (36% solvent B at t_G_ ≈ 30 min) for the columns with C_18_ stationary phases. For the less hydrophobic PS-DVB column, the end gradient composition needed to be significantly lower (20% solvent B at t_G_) to ensure t_final_ ≈ t_G_. With the flow rates investigated, no major effects on signal intensity were observed (two-fold changes or less, Supplementary Figure 5 & 6). The average relative standard deviations (RSDs) for the t_R_ in the ExSMix were 0.3, 0.2, 0.4 and 1.0% for the Chromolith, Accucore, PepMap and PepSwift, respectively. Carryover was negligible (<LOD) for all columns regarding the analytes and concentrations investigated in this study.

### Comparison of column performance, using complex samples

Using the optimized LC conditions for each column, and ddMS/MS acquisition, 17 proteotypic peptides (Figure 1) of proteins associated with the β-catenin degradasome were selected for further targeted MS/MS studies.

The effect of sample complexity-related retention time robustness (important for example MS/MS scheduling/aiding selectivity) was examined by comparing retention times of the simple ExSMix proteotypic peptides with that of the ExSMix spiked to tryptic HCT15 cell lysate (serving as a complex matrix). The median change in retention time of the peptides in the ExSMix and the ExSMix spiked cell sample was significantly larger for the monoliths (-2.1 and -1.6 min, for the organic and silica monolith, respectively) compared with the particle packed columns (-0.5 and -1.2 min, for the totally porous and solid core particle packed columns, respectively, Supplementary Figure 8). The average retention time RSDs of complex samples were 0.8 (1.9), 0.6 (1.0), 2.6 (2.8) and 1.3 (2.3)% (n ≥ 3) for the Chromolith, Accucore, PepMap and PepSwift, respectively (largest variation in parenthesis) (Figure 1). Figure 2 shows extracted ion chromatograms of the representative β-catenin peptide LLNDEDQVVVNK in each sample chromatographed on the four column set-ups. The peak widths did not change as the sample complexity increased (between ExSMix and ExSMix added to the cell lysate), indicating no sign of column overload.

### Comparison of targeted nanoLC-MS/MS performance: detecting Wnt proteins in colon cancer cells

The proteotypic peptides were subsequently searched for in an unspiked cell sample. For identification minimum three intense/descriptive MS/MS transitions were required. An additional criterion was that the retention time variation was maximum ±0.5 min relative to that of the ExSMix in spiked sample (matrix matching, see Supplementary Figure 9) to ensure very confident identification.

β-catenin (downstream target of Wnt/signaling) and GSK3β (a kinase crucial for N-terminal phosphorylation of β-catenin that leads to its degradation) (∼60 ng/µg and ∼15 ng/µg sample), respectively) were clearly identifiable with all the columns with the above-mentioned criteria. With the criteria employed the two other trace proteins (AXIN2, TNKS2, <1 ng/µg) were however not identified. Notably, using just two intense/descriptive MS/MS transitions would cause false positives in our assay; for example, the C-terminal HSSPSGTVAAR peptide of intact APC (not present in HCT15 colon cancer cells, due to a premature stop codon [26]) was falsely identified with otherwise same criteria (data not shown).

### Quantification with isotopically labeled internal standard

Simultaneous protein quantification with western blot (WB) is time consuming, specificity is antibody-dependent and values are often related to house-keeping proteins (e.g., Actin). LC-MS/MS is another alternative that allows for multiple proteins to be quantified with a high degree of specificity. For quantification in complex samples, isotopically labeled ISs are often added, either as peptides or proteins (e.g., AQUA peptides [27] or SILAC [19]). In this study, SILAC was used to label two cell lines that were pooled and used as an internal standard protein solution (IS_prot_) in following experiments (preparation and workflow described in Materials and methods and Supplementary Figure 10). In contrast to for example, spiking with single IS peptides, the labeled mix is a ‘universal’ IS, also provides correction for protein digestion, SPE clean-up and MS-response. Also the approach is simpler than producing recombinant-labeled IS proteins [28].

To see whether the solid core column (chosen due to best column performance, see above) would allow detecting changes in proteotypic peptide levels of β-catenin and GSK3β, we selected the colon carcinoma cell lines HCT15, COLO320DM and SW480 cells (IS_prot_ added). Each were treated with a selective tankyrase inhibitor (G007-LK [17]) and subjected to quantification with LC-MS/MS and WB. β-catenin (the subject of the AXIN2/tankyrase2/APC/GSKbeta-containing destruction complex) could be relatively quantified with excellent precision (RSD 8%) in these solutions with double complexity (sample + IS_prot_) well within 20 min: Reduction of β-catenin following treatment with the selective tankyrase inhibitor G007-LK was observed in SW480 cells and correlated with results obtained with an established WB protocol (see Figure 3 and [16]). For levels of GSK3β and β-catenin in the other cell lines analyzed, see Supplementary Figure 11.

## Discussion

We have compared some of the most common nanoLC columns/morphologies used for relatively fast, targeted proteomics, for determination of central proteins in the Wnt signal pathway. Using 30 min solvent gradients (t_M_ and t_G_ was similar for all columns after optimization), the solid core particle packed nanocolumn provided the highest peak capacity and hence resolution, with the silica monolith at a clear second place. Similar results have been observed with larger-bore (i.e., larger inner diameter) columns [29], but it was not given that this would be the case for nano-scale columns, as successful packing/polymerization can be dependent on column diameter. For example, solid core particles packed in 2.1 mm ID columns have previously not been able to match the efficiency of comparable 4.6 mm ID columns [30], while monolith columns are easier to prepare in capillary/nanoformat [31].

The column’s retention time robustness (of importance for strict identification and MS/MS scheduling) differed significantly, with the solid core column having the best within-complex sample repeatability. The retention time robustness of the solid core particle packed column was somewhat surprising considering its lower surface area. We believe this factor was less important due to the use of a full porous particle packed precolumn. The columns were rather similar regarding other traits such as carryover and loading capacity.

Although the solid core column had arguably the best performance regarding peak capacity, there were no differences in the number of target proteins/proteotypic peptides detected in complex samples. It is possible that the differences between today’s state-of-the-art columns are to a large degree insignificant when handling complex samples? Performing untargeted proteomics further supported this hypothesis; the number of identified proteins was virtually identical when using different commercial columns (see Supplementary Figure 12 and Supplementary Excel File).

## Conclusion

Is there convincing evidence that the choice of commercial nanoLC column will clearly affect success in relatively fast, targeted proteomics? Based on our case study of Wnt pathway proteins, our answer is ‘no’. To enable more sensitive targeted proteomics, we believe that more focus should be on developing very narrow columns (low µm ID); such columns are operated at low flow rates (low nl/min), which is associated with a very low degree of ion suppression [32].

## Future perspective

We believe that the field of proteomics will move to more hypothesis-driven research, with targeted methods being more important than the previous comprehensive approaches. Systems will probably develop into more plug-and-play solutions which enable labs to transfer from antibody-based techniques. Quick sample preparation and downscaling of columns will be important in the next years, as well as method specificity and robustness. We also believe that the mass spectrometers will have to be miniaturized in to fit the chromatographic systems.

**Table 1.** **Optimal flow rate, gradient composition, peak capacity, peak width and asymmetry based on data-dependent MS/MS scans of separated 1 ng ExSMix from recombinant APC, AXIN2, β-catenin, GSK3β and TNKS2 for the four column set-ups. Retention time relative standard deviation values are based on three injections, and for at least five peptides.**

**Column**	**Precolumn**		**Analytical column**		**Precolumn/analytical column material**	**Optimal flow rate (nl/min/linear velocity in cm/s)**	**Gradient end (% [v/v] ACN)**	**Retention time variation (RSD, %)**	**Peak capacity at half peak height (n ≥ 3; RSD < 10 %)**	**Peak width at half peak height (n ≥ 3; s)**	**Asymmetry (n ≥ 3)**
	**ID (µm)**	**Length (mm)**	**ID (µm)**	**Length (mm)**							
Chromolith^®^CapRod^®^	100	50	50	150	C18 silica monolithic/	200/0.8	36	0.3	130	19.8	1.4
					C18 silica monolithic						
PepMap™	75	20	50	150	C18, 3 µm, 100 Å particles/	200/0.9	36	0.4	106	16.2	1.1
					C18, 2 µm, 100 Å particles						
Accucore™	75	20	75	150	C18, 3 µm, 100 Å particles/	300/0.7	36	0.2	189	10.2	1.1
					C18, 2.6 µm, 80 Å solid core particles						
PepSwift™	200	50	100	250	PS-DVB monolithic/	500/0.5	20	1.0	100	16.2	1.5
					PS-DVB monolithic						

ACN: Acetonitrile.

**Executive summary**

Commercial nanoLC columns do not have significant performance difference for targeted determination of proteins in complex samples, even though they differ in chromatographic performance.
Solid core and porous particle packed columns compared with organic and silica monoliths are more robust in terms of retention time shift in standards versus complex samples.
Peak capacity does not affect the number of identified proteins in 30 min gradients.
Sensitivity is not drastically affected in the flow range of 500–200 nl/min.
Moderate abundant proteins; β-catenin and GSK3β can be quantified with low relative standard deviationsand correlate well to western blot results.
