# Supplementary material for: Comparison of commercial nanoliquid chromatography columns for fast, targeted mass spectrometry-based proteomics
Source: Future Sci OA. 2016 Mar 16;2(2):FSO119. doi: 10.4155/fsoa-2016-0014 (PMC5137844; doi:10.4155/fsoa-2016-0014)
Supplement: Supplementary file 1 [file fsoa-02-119-s1.docx]

**Supplementary information**

Supplementary Materials and Methods

**Western blotting**

To 20 μL sample, 5 μL 5x SDS-loading buffer (containing 0.25 % (w/v) bromophenol blue (Sigma Aldrich), 0.5 M DTT (Sigma), 50 % (v/v) glycerol (Sigma Aldrich), 10 % (w/v) SDS (Sigma Aldrich) and 0.25 M Tris-HCl (Oslo University Hospital)) was added and the samples were denaturated for 15 min in a heating block set at 70°C. Gel separation was performed at constant voltage (PowerPAC HC Power Supply, Bio-Rad Laboratories Inc.) on a NuPAGE® Novex 3-8 % Tris-Acetate Gel (1.0 mm, 10 Well, Invitrogen™) for approximately 1 hour in a Tris-acetate running buffer (NuPAGE® Tris-Acetate SDS Running buffer 20X, Invitrogen) diluted to a working concentration of 1X.

Proteins were subsequently transferred to a polyvinyllidene fluoride membrane (PVDF, Immobilon®-P, Millipore) between two Whatman-papers (Bio-Rad Laboratories Inc) using a Trans-Blot SD Semi-Dry Electrophoretic transfer cell (Bio-Rad Laboratories Inc.) o/n in 4°C, at 50 mA per/gel. Prior to transfer, the PVDF-membrane was soaked according to manufacturers protocol. Blocking of the membrane was done for 8 hours in 4°C in blocking buffer containing 5% (w/v) non-fat milk (AppliChem GmbH, Darmstadt, Germany) in 1X PBS-Tween solution (PBS-Tween tablets, Medicago., Quebec City, QC, Canada). Primary antibody of beta-catenin (mouse anti-beta-catenin, BD Transductions Laboratories, CA, USA)) was added to 10 mL of blocking buffer to a final concentration of 1 µg/mL together with 1:4000 (*v/v*) anti α-actin antibody (Sigma Aldrich) and incubated o/n at 4°C with slow agitation. After incubation o/n, the PVDF-membrane was washed with PBS-T-solution 6 x 10 minutes before adding HRP-rabbit antibody (mouse anti-rabbit IgG-HRP: sc-2357, Santa-Cruz Biotechnology Inc.) dissolved in blocking buffer to a final ratio of 1:2000 (v:v), and incubated at room temperature with slow agitation for 2 hours. Prior to incubation with detection reagent (ECL Prime Western Blotting Detection Reagents, Amersham, GE Healthcare), the PDVF-membrane was washed with PBS-T.

Supplementary tables

Supplementary Table 1 – Amino acid sequence of selected proteins

| Amino acid sequence of recombinant proteins | |
| --- | --- |
| APC | NPVPVSETNESSIVERTPFSSSSSSKHSSPSGTVAARVTPFNYNPSPRKSSADSTSARPSQIPTPVNNNTKKRDSKTDSTESSGTQSPKRHSGSYLVTSV |
| AXIN2 | EDHKEPKKLAGVHALQASELVVTYFFCGEEIPYRRMLKAQSLTLGHFKEQLSKKGNYRYYFKKASDEFACGAVFEEIWEDETVLPMYEGRILGKVERID |
| beta-catenin | MSPILGYWKIKGLVQPTRLLLEYLEEKYEEHLYERDEGDKWRNKKFELGLEFPNLPYYIDGDVKLTQSMAIIRYIADKHNMLGGCPKERAEISMLEGAVLDIRYGVSRIAYSKDFETLKVDFLSKLPEMLKMFEDRLCHKTYLNGDHVTHPDFMLYDALDVVLYMDPMCLDAFPKLVCFKKRIEAIPQIDKYLKSSKYIAWPLQGWQATFGGGDHPPKSDLEVLFQGPLGSATQADLMELDMAMEPDRKAAVSHWQQQSYLDSGIHSGATTTAPSLSGKGNPEEEDVDTSQVLYEWEQGFSQSFTQEQVADIDGQYAMTRAQRVRAAMFPETLDEGMQIPSTQFDAAHPTNVQRLAEPSQMLKHAVVNLINYQDDAELATRAIPELTKLLNDEDQVVVNKAAVMVHQLSKKEASRHAIMRSPQMVSAIVRTMQNTNDVETARCTAGTLHNLSHHREGLLAIFKSGGIPALVKMLGSPVDSVLFYAITTLHNLLLHQEGAKMAVRLAGGLQKMVALLNKTNVKFLAITTDCLQILAYGNQESKLIILASGGPQALVNIMRTYTYEKLLWTTSRVLKVLSVCSSNKPAIVEAGGMQALGLHLTDPSQRLVQNCLWTLRNLSDAATKQEGMEGLLGTLVQLLGSDDINVVTCAAGILSNLTCNNYKNKMMVCQVGGIEALVRTVLRAGDREDITEPAICALRHLTSRHQEAEMAQNAVRLHYGLPVVVKLLHPPSHWPLIKATVGLIRNLALCPANHAPLREQGAIPRLVQLLVRAHQDTQRRTSMGGTQQQFVEGVRMEEIVEGCTGALHILARDVHNRIVIRGLNTIPLFVQLLYSPIENIQRVAAGVLCELAQDKEAAEAIEAEGATAPLTELLHSRNEGVATYAAAVLFRMSEDKPQDYKKRLSVELTSSLFRTEPMAWNETADLGLDIGAQGEPLGYRQDDPSYRSFHSGGYGQDALGMDPMMEHEMGGHHPGADYPVDGLPDLGHAQDLMDGLPPGDSNQLAWFDTDL |
| GSK3β | MSGRPRTTSFAESCKPVQQPSAFGSMKVSRDKDGSKVTTVVATPGQGPDRPQEVSYTDTKVIGNGSFGVVYQAKLCDSGELVAIKKVLQDKRFKNRELQIMRKLDHCNIVRLRYFFYSSGEKKDEVYLNLVLDYVPETVYRVARHYSRAKQTLPVIYVKLYMYQLFRSLAYIHSFGICHRDIKPQNLLLDPDTAVLKLCDFGSAKQLVRGEPNVSYICSRYYRAPELIFGATDYTSSIDVWSAGCVLAELLLGQPIFPGDSGVDQLVEIIKVLGTPTREQIREMNPNYTEFKFPQIKAHPWTKDSSGTGHFTSGVRVFRPRTPPEAIALCSRLLEYTPTARLTPLEACAHSFFDELRDPNVKLPNGRDTPALFNFTTQELSSNPPLATILIPPHARIQAAASTPTNATAASDANTGDRGQTNNAASASASNST |
| TNKS2 | MHHHHHHSSGVDLGTENLYFQSMLNTSGSGTILIDLSPDDKEFQSVEEEMQSTVREHRDGGHAGGIFNRYNILKIQKVCNKKLWERYTHRRKEVSEENHNHANERMLFHGSPFVNAIIHKGFDERHAYIGGMFGAGIYFAENSSKSNQYVYGIGGGTGCPVHKDRSCYICHRQLLFCRVTLGKSFLQFSAMKMAHSPPGHHSVTGRPSVNGLALAEYVIYRGEQAYPEYLITYQIMRPEG |

**Supplementary Table 2 – Precursor and fragment *m/z* of monitored peptides**

| **Protein** | **Peptide** | **Precursor *m/z*** | **Fragment**  ***m/z*** | | | | | | |
| --- | --- | --- | --- | --- | --- | --- | --- | --- | --- |
| **APC** | HSGSYLVTSV | [M+2H]^2+^  525.26703 | b_5_^+^  532.2155 | b_6_^+^  645.2994 | b_7_^+^  744.3679 | b_8_^+^  845.4154 |  |  |  |
|  | HSSPSGTVAAR | [M+2H]^2+^  535.27283 | y_7_^+^  661.3628 | y_8_^+^  758.4164 | y_9_^+^  845.4484 | y_10_^+^  932.4807 |  |  |  |
|  | VTPFNYNPSPR | [M+2H]^2+^  646.32483 | y_4_^+^  456.2569 | y_9_^2+^  546.2677 | y_7_^+^  733.3632 | y_7_^+^  847.4062 | y_8_^+^  994.475 | y_9_^+^  1091.528 |  |
| **AXIN2** | AQSLTLGHFK | [M+2H]^2+^ 551.30621 | y_3_^+^  431.2406 | y_4_^+^  488.2612 | y_6_^+^  702.3938 | y_7_^+^  815.4778 | y_9_^+^  902.51 |  |  |
|  | ILGKVER | [M+2H]^2+^  407.76141 | y_6_^2+^  351.2193 | y_4_^+^  531.3258 | y_5_^+^  588.3472 | y_6_^+^  701.4313 |  |  |  |
|  | ILGKVERID | [M+2H]^2+^  521.81732 | y_7_^2+^  408.7329 | y_8_^2+^  465.2752 | y_4_^+^  532.2734 | y_5_^+^  631.3419 | y_6_^+^  759.4373 | y_7_^+^  816.4584 | y_8_^+^  929.54254 |
| **Beta-catenin** | LLNDEDQVVVNK | [M+2H]^2+^  693.36731 | y_4_^+^  459.2929 | y_7_^+^  801.447 | y_8_^+^  930.4899 | y_9_^+^  1045.5161 | y_10_^+^  1159.5590 |  |  |
|  | HAVVNLINYQDDAELATR | [M+2H]^2+^ 681.35327 | y_8_^+^  890.4222 | y_9_^+^  1018.481 | y_10_^+^  1181.544 | y_11_^+^  1295.588 |  |  |  |
|  | NEGVATYAAAVLFR | [M+2H]^2+^ 741.39801 | y_7_^+^  747.4519 | y_8_^+^  910.5156 | y_19_^+^  1011.563 | y_10_^+^  1082.6 |  |  |  |
|  | ATVGLIR* | [M+2H]^2+^ 365.2345 | y_4_^+^  458.309 | y_5_^+^  557.3776 |  |  |  |  |  |
| **GSK3beta** | DIKPQNLLLDPDTAVLK | [M+2H]^2+^  947.54236 | y_5_^+^  531.3515 | y_7_^+^  743.4316 | y_9_^+^  971.5436 | y_10_^+^  1084.628 | y_12_^+^  1362.727 | y_14_^+^  1536.867 |  |
|  | DSSGTGHFTSGVR | [M+2H]^2+^ 654.30316 | y_4_^+^  418.2413 | y_5_^+^  519.2893 | y_8_^+^  860.4382 | y_9_^+^  961.486 | y_10_^+^  1018.508 |  |  |
|  | LLEYTPTAR | [M+2H]^2+^ 532.2926 | y_4_^+^  444.2571 | y_5_^+^  545.3049 | y_6_^+^  708.3682 | y_7_^+^  837.411 | y_8_^+^  950.4953 |  |  |
|  | VIGNGSFGVVYQAK | [M+2H]^2+^  719.88977 | y_4_^+^  509.2725 | y_5_^+^  608.3408 | y_7_^+^  764.4305 | y_8_^+^  911.4993 | y_10_^+^  1055.553 | y_13_^+^  1226.618 |  |
| **TNKS2** | DGGHAGGIFNR | [M+2H]^2+^  550.76611 | y_10_^2+^  493.2524 | y_5_^+^  606.3369 | y_6_^+^  663.3583 | y_7_^+^  734.3954 |  |  |  |
|  | EVSEENHNHANER | [M+3H]^3+^  522.2298 | y_y3_^+^  418.205 | y_4_^+^  495.8343 | y_10_^2+^  626.3012 | y_11_^2+^  668.7856 | y_6_^+^  740.3443 | y_7_ ^+^  877.4038 |  |
|  | SFLQFSAMK | [M+2H]^2+^  529.76984 | y_5_^+^  583.2908 | y_6_^+^  711.3494 | y_7_^+^  824.4333 |  |  |  |  |

**Supplementary Figures**

Supplementary Figure 1 – Peak capacity at half peak height and peak symmetry (asymmetry) at 10% peak height calculated based on > 5 proteotypic peptides for the set-up consisting of a 75 μm x 20 mm PepMap™ pre-column connected to a 50 μm x 150 mm PepMap™ analytical column at various flow-rates(n=1). A 1 ng sample containing tryptic peptides from AXIN2, APC, beta-catenin, GSK3beta and TNKS2 was chromatographed using gradient elution from 3 to 36% (ACN/0.1%FA) in 30 minutes.

Supplementary Figure 2 - Peak capacity at half peak height and peak symmetry (asymmetry) at 10% peak height calculated based on > 5 proteotypic peptides for the set-up consisting of a 100 μm x 50 mm Chromolith® CapRod® precolumn connected to a 50 μm x 150 mm Chromolith® CapRod® analytical column at various flow-rates (n=1). A 1 ng sample containing tryptic peptides from AXIN2, APC, beta-catenin, GSK3beta and TNKS2 was chromatographed using gradient elution from 3 to 36% (ACN/0.1%FA) in 30 minutes.

Supplementary Figure 3 - Peak capacity at half peak height and peak symmetry (asymmetry) at 10% peak height calculated based on > 5 proteotypic peptides for the set-up consisting of a 200 μm x 50 mm PepSwift™ precolumn connected to a 100 μm x 250 mm PepSwift™ analytical column at various flow-rates (n=1). A 1 ng sample containing tryptic peptides from AXIN2, APC, beta-catenin, GSK3beta and TNKS2 was chromatographed using gradient elution from 3 to 36% (ACN/0.1%FA) in 30 minutes.

Supplementary Figure 4 - Peak capacity at half peak height and peak symmetry (asymmetry) at 10% peak height calculated based on > 5 proteotypic peptides for the set-up consisting of a 75 μm x 20 mm PepMap™ precolumn connected to a 50 μm x 150 mm PepMap™ analytical column at various flow-rates (n=1). A 1 ng sample containing tryptic peptides from AXIN2, APC, beta-catenin, GSK3beta and TNKS2 was chromatographed using gradient elution from 3 to 36% (ACN/0.1%FA) in 30 minutes. **Peak capacity obtained with optimal gradient condition, from 3 to 20% (ACN/0.1%FA) in 30 minutes.

Supplementary Figure 5 - Relative peak areas with standard deviation calculated from EICs of > 5 proteotypic peptides for each column studied based on optimal separation conditions (n=3) (see Materials and Methods). SilicaTip emitters were used for the Accucore™ and PepSwift™ columns (black) Stainless steel emitters were used for the Chromolith®CapRod® and PepMap™ columns (grey).

Supplementary Figure 6 - Peak capacity at half peak height, relative peak volume and peak area calculated based on > 5 proteotypic peptides for the set-up consisting of a 100 μm x 50 mm Chromolith® CapRod® precolumn connected to a 50 μm x 150 mm Chromolith® CapRod® analytical column at various flow-rates (n=1). A 1 ng sample containing tryptic peptides from AXIN2, APC, beta-catenin, GSK3beta and TNKS2 was chromatographed using gradient elution from 3 to 36% (ACN/0.1%FA) in 30 minutes.


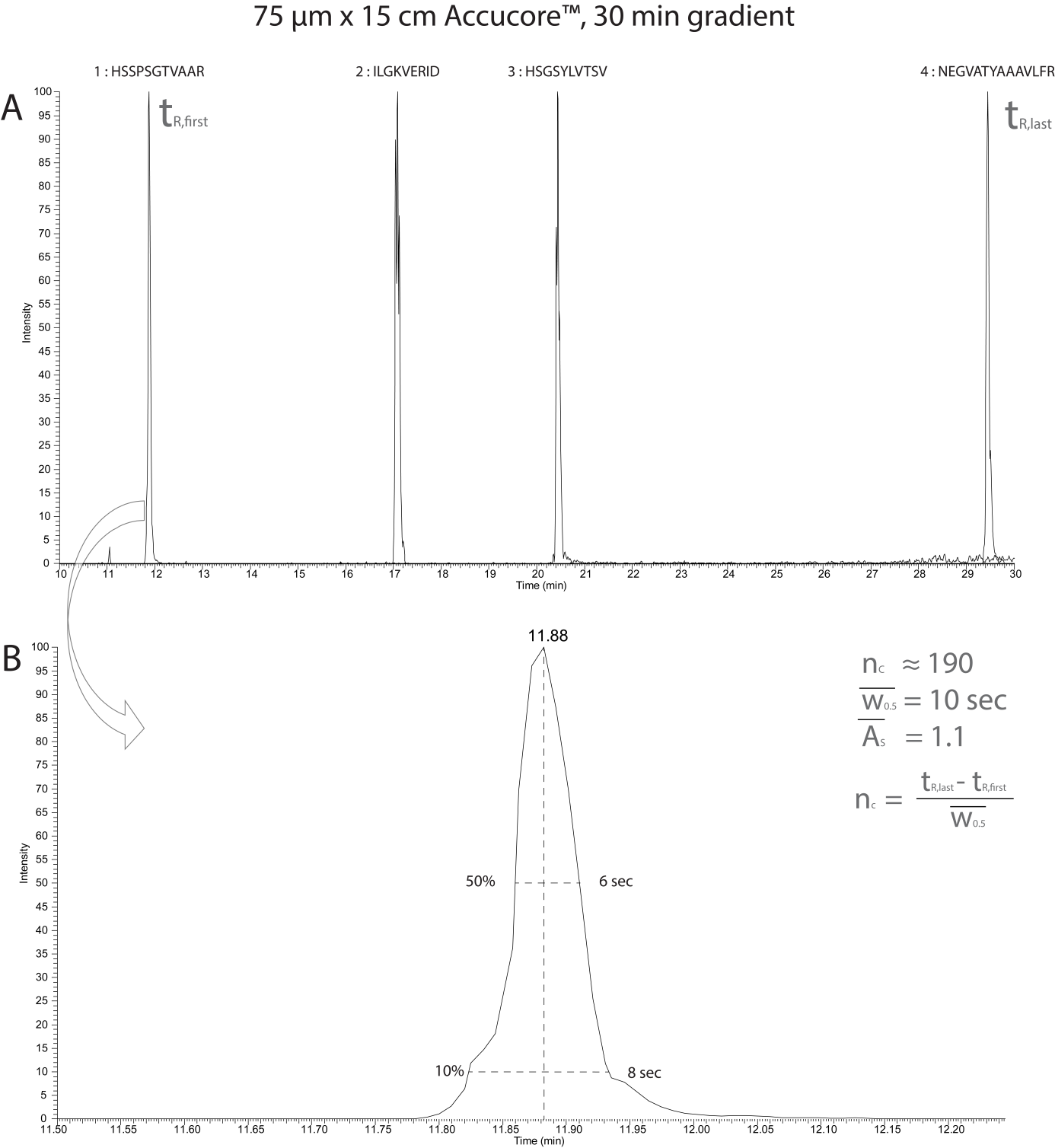


**Supplementary Figure 7** - A : EIC of peptides from 1 ng standard tryptic mixture chromatographed using the 75 µm x 15 cm Accucore™ solid core particle packed column set-up. B : EIC of HSSPGTVAAR with peak widths at 10 % and 50 % peak height and overall peak capacity n_C_, average peak width ( $\bar{w_{0.5}}$ ) and average asymmetry factor ($\bar{A_{S}}$).

**Supplementary Figure 8 -** Retention time shift in ExSMix spiked in standard versus ExSMix for a set of peptides chromatographed at optimal conditions (See **Methods and Materials**). The vertical bar is median value.


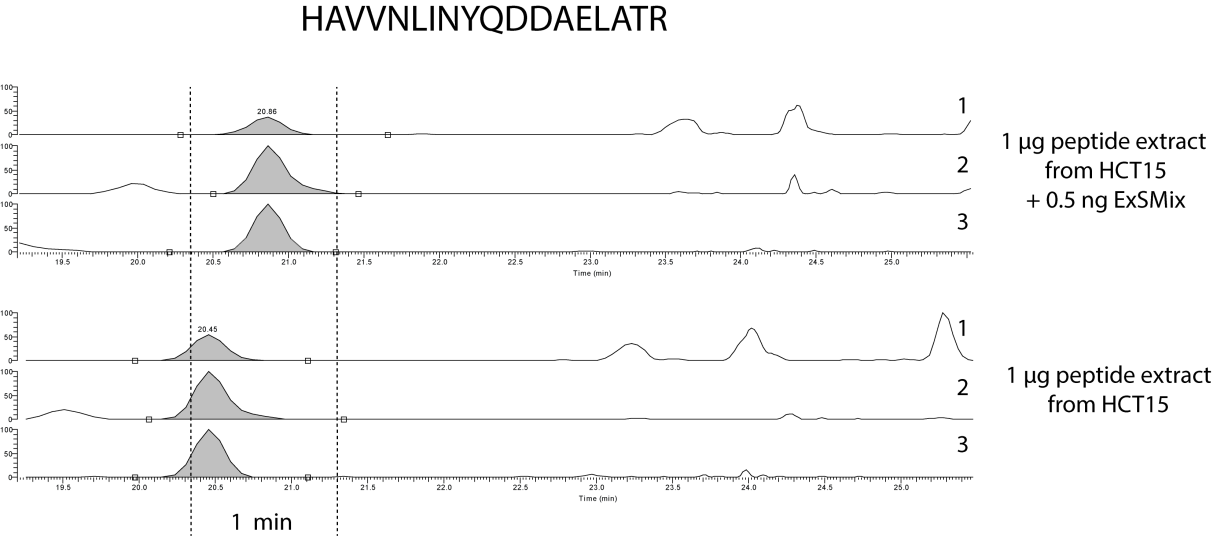


Supplmentary Figure 9 – EIC of the high-abundance peptide (HAVVNLINYQDDAELATR – beta-catenin), with transitions from *m/z* = 1021.51898 to 1) *m/z* = 775.3958, 2) *m/z* = 1181.545, 3) *m/z* = 1295.588 in spiked and unspiked tryptic digested HCT15 extract. Grey background equals positive identification. Samples were chromatographed on the Accucore™ column set-up with parameters as described in Materials and Methods.


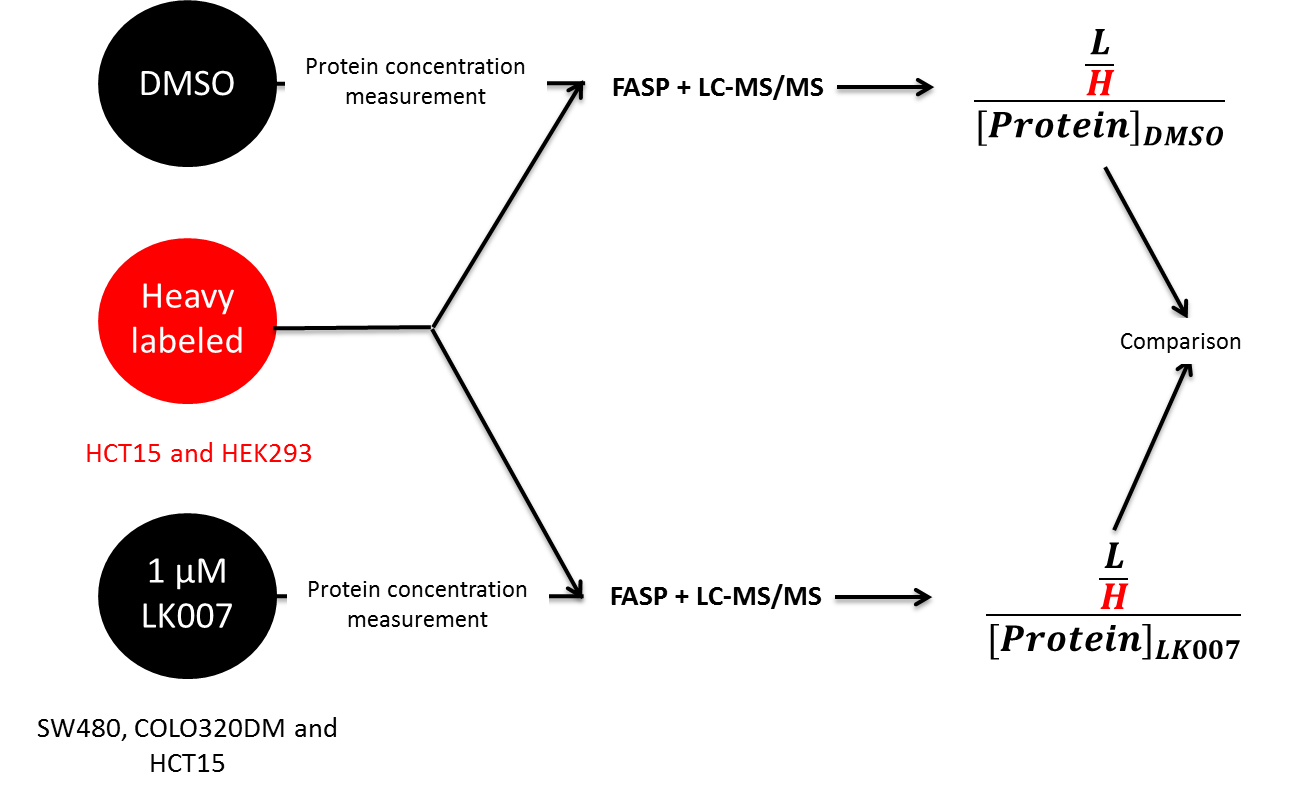


**Supplementary Figure 10** – Sample processing outline for quantification showing normalization of L/H ratio to protein concentration. See **Methods and Materials** for more information.

**Supplementary Figure 11** - Relative concentration of beta-catenin and GSK3beta measured in HCT15, COLO320DM and SW480 cells treated with DMSO and 1 µM LK007 for 24 hours, respectively. Ratios are relative to original protein concentration after cell lysis (**Supplementary Figure 12**). The p-values were based on 3 biological replicates with exception of SW480 1 µM LK007, with 2 replicates . LC-MS/MS analysis was performed on the Accucore™ column set-up with standard gradient conditions and optimized parameters as described in **Materials and Methods**.

**Supplementary Figure 12 –** Number of identifications (n=2) with 120 minute gradient on the PepMap and Accucore columns with data-dependent MS/MS acquisition of tryptic extract of SILAC labeled HCT15 cells. A minimum of 2 peptides per protein, where at least 1 is unique were required for identification. MS conditions as described in Materials and Methods. The vertical bar is the average of the two injections.
